# Fecal microbial transplantation as a novel therapeutic for autism spectrum disorders: a review of the current literature

**DOI:** 10.3389/frmbi.2023.1222089

**Published:** 2023-08-15

**Authors:** Rebecca Gudka, Iveren Winifred Nyinoh

**Affiliations:** ^1^ College of Medicine and Health, University of Exeter, Exeter, United Kingdom; ^2^ Department of Biological Sciences, Benue State University, Makurdi, Nigeria

**Keywords:** fecal microbial transplantation, gut microbiome, gut microbiota, microbiomegut-brain axis, autism spectrum disorder

## Abstract

**Background:**

Autism spectrum disorders (ASDs) are complex neurobiological conditions with poor long-term outcomes and limited treatment options. The microbiota–gut–brain axis indicates a pathway by which the gut microbiota links to ASDs. Fecal microbial transplantation (FMT), whereby the gut microbiota is replaced with that of a healthy individual, shows promise for the treatment of neurobiological conditions. This review examines the current evidence for the use of FMT as a therapeutic for ASD.

**Discussion:**

ASDs and their associated gastrointestinal symptoms are improved with FMT, potentially due to the engraftment of features of a healthy gut. Longer treatment regimens that include daily maintenance doses appear to be the most effective long-term therapeutic option, with benefits persisting 2 years post-intervention. Evidence is mixed regarding the use of preparatory treatments. Considering the sex bias in ASD research, small sample sizes and the lack of placebo control arms, randomized controlled trials would be of benefit to the evidence base regarding the use of FMT as a therapeutic option for ASD.

**Conclusion:**

FMT is a promising new therapeutic for ASD, but the evidence base is in its infancy.

## 1 Introduction

Autism spectrum disorders (ASDs) are a complex and highly heterogeneous set of neurobiological disorders. Despite the rising prevalence of ASD in children—currently estimated to be 1 in 100 worldwide—its etiology is poorly understood (Chawner et al., 2021; Zeidan et al., 2022). However, evidence suggests that genetic and environmental factors, immunomodulatory disorders, inflammation, and the gut microbiome may all affect the onset and development of ASD (Cheroni et al., 2020; Han et al., 2021).

Individuals with ASD experience symptoms that vary in severity from one another and over time. Seltzer et al. (2003) described the symptoms in children and adults who had been clinically diagnosed with ASD. The symptoms include loss of skills over time, while also noting that ASD plateaus in certain individuals in adolescence but continues to develop into adulthood in others. The *Diagnostic and Statistical Manual of Mental Disorders*, Fifth Edition (DSM-5, 2013), as cited in Young et al. (2020), describes the core symptoms used to identify individuals with ASD, which include difficulties with verbal communication, impaired social interaction, restricted interests, and repetitious behavior. Overall, the symptoms of ASD often persist into adulthood; negative long-term outcomes include a dependence on parents, social and economic limitations, and additional mental health conditions (Chamak and Bonniau, 2016). Effective pharmacological and psychological/behavioral interventions for core symptoms of ASD are limited and can cause significant negative side effects (Carter et al., 2011; Kent et al., 2013; Marraffa and Araba, 2016). The increasing rates of diagnoses and a lack of effective therapeutic options highlight the importance of investigating alternative treatments for ASD.

Significant gastrointestinal (GI) symptoms often co-occur in children and adults with ASD; ASD severity correlates with GI symptoms such as constipation, diarrhea, and indigestion (Babinska et al., 2020). ASD and GI issues may be linked to dysbiosis in the gut microbiota *via* the microbiota–gut–brain axis: a bidirectional connection between the microbiota, the gut, and the brain (Li and Zhou, 2016). Children with ASD have significantly distinct gut microbiota profiles from those of typically developing (TD) children, with a significantly lower level of microbial diversity and greater abundance of *Clostridium* and *Bacteroides* found in the gut microbiota of children with ASD (Chen et al., 2020; Qureshi et al., 2020). This is of significance because metabolites such as propionic acid (PPA), which is produced by *Clostridium* and *Bacteroides*, have been shown to induce autistic-like behaviors in mice (Shultz et al., 2015). In addition, *Clostridium histolyticum* can result in symptoms and behaviors of ASD by inhibiting the release of certain neurotransmitters due to the production of a neurotoxin that enters the central nervous system (Finegold et al., 2017). In light of this evidence, which shows that ASD behaviors may be a result of the microbial species that colonize the gut and their associated metabolites, the gut microbiota provides a suitable target for a therapeutic intervention for ASD.

Fecal microbial transplantation (FMT) is the delivery of a whole microbiota from a healthy donor into the GI tract of patients to reconstruct the gut microbiota (National Health Service, 2021). It is commonly used to alleviate recurrent *Clostridium difficile* infections, inflammatory bowel diseases, and ulcerative colitis (Brandt et al., 2012; Kunde et al., 2013; Allegretti et al., 2021). Clostridium-targeting oral vancomycin has previously been shown to temporarily ameliorate ASD symptoms, but the benefits were not sustained in the long term, and it may not fully eradicate the bacterial spores that can lead to recurrent C. difficile infections (Buggy, 1987; Sandler et al., 2016). In addition, pre- and probiotics have been shown to ameliorate specific symptoms of ASDs but not overall disease severity, and the clinical evidence is mixed (Tan et al., 2021). Pre- and probiotics are limited to only introducing a small number of bacterial species cultured from a non-human origin (Tan et al., 2021). FMT has the potential to overcome these barriers by introducing a diverse bacterial population from a TD individual. *C. difficile* is usually treated using just one dose of FMT; however, evidence suggests that for chronic conditions, such as ulcerative colitis, longer, more intensive treatment regimens confer better treatment (Borody, 2003; Kunde et al., 2013). Thus, the dose, treatment regimen, and administration route are of interest when determining the effect of FMT on ASD symptoms.

The aim of the present study was to summarize the findings and critically appraise the current research on the safety and efficacy of FMT as a therapeutic for core behavioral and related symptoms of ASD by answering the following research questions:

- Can FMT alter the gut microbiota and treat core behavioral symptoms of ASD?
- In which treatment regimen is FMT the most safe and effective for treating symptoms of ASD?

## 2 Methods and results

The research was carried out by conducting a search in PubMed® (National Library of Medicine, Bethesda, MD, USA) and Google Scholar (Google Inc., Mountain View, CA, USA) on 21 November 2022. The search terms used were ((faecal transplant*) OR (fecal transplant*) OR (fecal transfer) OR (microbiota transfer therapy) OR (microbiota transplant) OR (fmt) OR (fecal microbiota transplant*) OR (faecal microbiota transplant*)) AND ((autism) OR (autistic) OR (asd) OR (autistic spectrum disorder) OR (Autism spectrum disorder) OR (autism spectrum condition) OR (asc)).

The results were limited to publications between 2015 and the search date, and only full-text English-language papers were considered. The titles and abstracts were screened for relevance. Full-text reviews of the remaining papers were conducted. To be included, papers had to meet broad inclusion criteria: (1) participants had a diagnosis of ASD or represented an ASD model in non-human subjects, (2) participants were treated with FMT, and (3) the study measured the effect of FMT on behavioral symptoms of ASD. Finally, backward citation searching of a recent systematic review on the topic was conducted.

A total of 133 papers were yielded from the search, of which 123 were available in full-text and English-language formats. Thirty-one were found to be relevant after title and abstract screening, and 12 matched the inclusion criteria. Two additional studies were found through backward citation searching, but they were only available as conference abstracts, which were not deemed reliable because there were no reported data to verify the conclusions. **Table 1** shows a summary of the included studies.

**Table 1:** Characteristics of the studies included from the search results.

Study	ASD model/trial design	Number of participants (control: intervention)	Age range	Number of males (%)	Comorbid GI symptoms	Intervention
Abuaish et al. (2021)	Rat—propionic acid	28 (7: 21)	–	28 (100)	–	Rat → rat FMT
Abuaish et al. (2022)	Rat—propionic acid	28 (7: 21)	–	28 (100)	–	Rat → rat FMT
Abujamel et al. (2022)	Rat—propionic acid	20 (5:15)	–	20 (100)	–	Rat → rat FMT
Chen et al. (2020)	Mice—maternal immune activation	Not reported	–	Not reported	–	Human → rat FMT
Goo et al. (2020)	Mice—*Fmr1* KO mice	25 (8: 17)	–	16 (64)	–	Mice → mice FMT
Li et al. (2020)	Mice—EphB6 deficient	Not reported	–	– (100)	–	Mice → mice FMT
Kang et al. (2017)	Human—open-label clinical trial	38 (20: 18)	7–16 years	34 (89)	Yes	Human → human FMT
Kang et al. (2019)	Human—follow-up study	18 (0: 18)	9–18 years	16 (89)	Yes	Human → human FMT
Li et al. (2021)	Human—open-label clinical trial	56 (16: 40)	3–17 years	53 (95)	Yes	Human → human FMT
Pan et al. (2022)	Human—retrospective study	42 (0: 42)	3.75–8.25 years	34 (81)	Yes	Human → human FMT
Qureshi et al. (2020)	Human—secondary data analysis	38 (20: 18)*	7–16 years*	34 (89)*	Yes*	Human → human FMT*
Zhang et al. (2022)	Human—retrospective study	49 (25: 24)**	3–14 years	41 (84)	No	Human → human FMT

*Analysis of data derived from Kang et al. (2017).

**Both groups had ASDs and received the intervention; the control group had no constipation, whereas the intervention group had constipation. ASD, autism spectrum disorder; GI, gastrointestinal; FMT, fecal microbial transplantation.

Update searches in PubMed and Google Scholar were run on 2 May 2023, with no further studies found for inclusion in this article.

## 3 Discussion

### 3.1 Recent studies evaluating the effectiveness of fecal microbial transplantation therapy in autism spectrum disorders

Studies demonstrate that FMT significantly improves behavioral symptoms of ASD. Two open-label clinical trials and two retrospective studies found that post-intervention with FMT, ASD severity was significantly reduced relative to baseline when measured using the Childhood Autism Rating Scale (CARS) (Kang et al., 2017; Kang et al., 2019; Li et al., 2021; Pan et al., 2022; Zhang et al., 2022). The CARS is conducted by a professional evaluator and has previously been reported as a valid diagnostic tool with good sensitivity and specificity (Chlebowski et al., 2010). Kang et al. found that at both the 8-week and 2-year follow-ups there were no signs of reversal of the improvements to ASD symptoms, (Kang et al., 2017; Kang et al., 2019); 2 years after intensive treatment with FMT, only 17% children were rated as having severe ASD, compared with 83% at baseline, and 44% children scored below the ASD diagnostic threshold (Kang et al., 2019). This demonstrates the potential of FMT as a therapeutic with long-term benefits for children with severe ASD.

**Figure 1:**
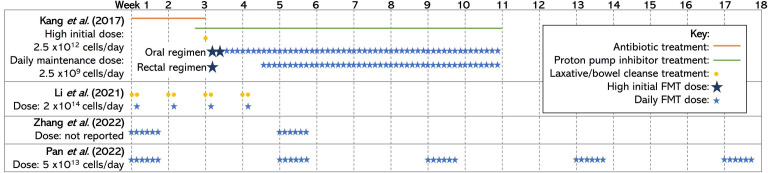
A diagram of the treatment regimens used by each included human study. The studies vary with their use of preparatory procedures such as antibiotic treatment, proton pump inhibitors, bowel clenses, and the size and number of doses/courses of FMT delivered. The information included in this figure has been obtained/adapted from (Kang et al., 2017; Zhang et al., 2022; Pan et al., 2022; Li et al., 2021) FMT, fecal microbial transplantation.

In contrast, Li et al. found that improvements in social skills deficits post-intervention were reversed at the 8- to 12-week follow-up in an open-label study of 40 children with ASD and GI problems (Li et al., 2021). A possible explanation for the differences in the findings is that different doses and treatment regimens were used. Kang et al. opted for a high initial dose of FMT followed by daily maintenance doses for 8 weeks, whereas Li et al. delivered a high dose once a week for 4 weeks (Kang et al., 2017; Li et al., 2021). **Figure 1** shows the treatment regimens used in human trials. Two additional studies also demonstrated that the effect of FMT on the severity of ASD symptoms increased with increasing treatment courses (Pan et al., 2022; Zhang et al., 2022). This evidence suggests that prolonged treatment, or multiple courses, may achieve longer-lasting benefits for children with ASD.

Overall, there were no reported severe adverse reactions to FMT, which suggests that it is a safe and well-tolerated treatment. However, Kang et al. found that 5%, 39%, and 28% of participants suffered from mild to moderate rashes, hyperactivity, and tantrums/aggression, respectively, when treated with vancomycin treatment prior to FMT (Kang et al., 2017). The use of preparation procedures in the studies varied (see **Figure 1**). The preparation procedures for FMT often include vancomycin, proton pump inhibitors, and bowel cleansing to prepare the intestinal landscape for engraftment of the donor microbiota (Kang et al., 2017). The effects of preparatory procedures on the safety and efficacy of FMT must be considered as previous research highlights the role of vancomycin and proton pump inhibitors on recipient microbiota, but they can result in negative side effects for participants (Vrieze et al., 2014; Freedberg et al., 2015; Sandler et al., 2016). Future research should compare the effects of FMT with and without preparatory procedures to determine whether it is necessary for FMT success. No differences on treatment safety or therapeutic outcome have been noted between receiving doses of FMT *via* oral or rectal administration in studies that used both administration routes (Kang et al., 2017; Li et al., 2021). Therefore, the ability to swallow capsules and patient/parent preference should be the determining factors when deciding between administration routes.

Two rodent studies found that social deficits in ASD mice were normalized upon treatment with FMT (Goo et al., 2020; Li et al., 2020); another three studies using rodents found that there was an increasing trend in social behavior upon treatment with FMT, although these findings did not reach significance (Chen et al., 2020; Abuaish et al., 2021; Abuaish et al., 2022). While these findings support the results from human trials, a criticism of using chemical- or immune-activated rodent models of ASD is that they are unlikely to simulate the complex interplay between pathophysiology, genetics, and environmental factors that humans experience. This is supported by Bronfenbrenner’s ecological systems theory, which describes the effect of the external environment on a child’s development, including in those children with developmental disorders (Bronfenbrenner, 1979).

### 3.2 Association between gastrointestinal symptoms and autism spectrum disorder severity

FMT significantly improves GI symptoms for children with ASD (Kang et al., 2017; Kang et al., 2019; Li et al., 2021; Pan et al., 2022; Zhang et al., 2022). Indigestion, diarrhea, abdominal pain, and constipation were all significantly improved relative to baseline across multiple studies, alongside a reduction in the number of days where participants experienced abnormal or no stools (Kang et al., 2017; Kang et al., 2019; Li et al., 2021; Pan et al., 2022). These improvements persisted at the 2-year follow-up (Kang et al., 2019). The presented findings are not surprising considering the evidence for the use of FMT to treat isolated GI health conditions (Borody, 2003; Kunde et al., 2013; Allegretti et al., 2021).

The Gastrointestinal Symptom Rating Scale (GSRS) is a disease-specific questionnaire based on 15 elements, divided into five symptom groups that describe GI symptoms such as reflux, abdominal pain, indigestion, diarrhea, and constipation. According to the GSRS, symptoms are rated from one to seven on a Likert-type scale, where one represents no troublesome symptoms and seven represents very distressing symptoms (Kulich et al., 2008). Kang et al. found a significant positive correlation between the percentage changes in GSRS scores and CARS scores (Kang et al., 2019). This is supported by previous research that shows a link between GI and ASD severity, but from these data it was not possible to ascertain a causative effect (Babinska et al., 2020). In addition, Zhang et al. found a greater positive effect on ASD severity in children who were not constipated than for those who were constipated prior to FMT (Zhang et al., 2022). The authors suggested that, in children who have pre-existing GI symptoms, FMT may better improve ASD symptom severity if the children’s GI problems are treated first. Apart from one study, all human trials used only participants who had co-occurring GI issues alongside ASD (see **Table 1**). This creates a gap in the research, with more evidence required to evaluate whether ASD symptoms would improve to a greater extent if there were no co-occurring GI symptoms.

### 3.3 Restructuring the microbiota

**Figure 2:**
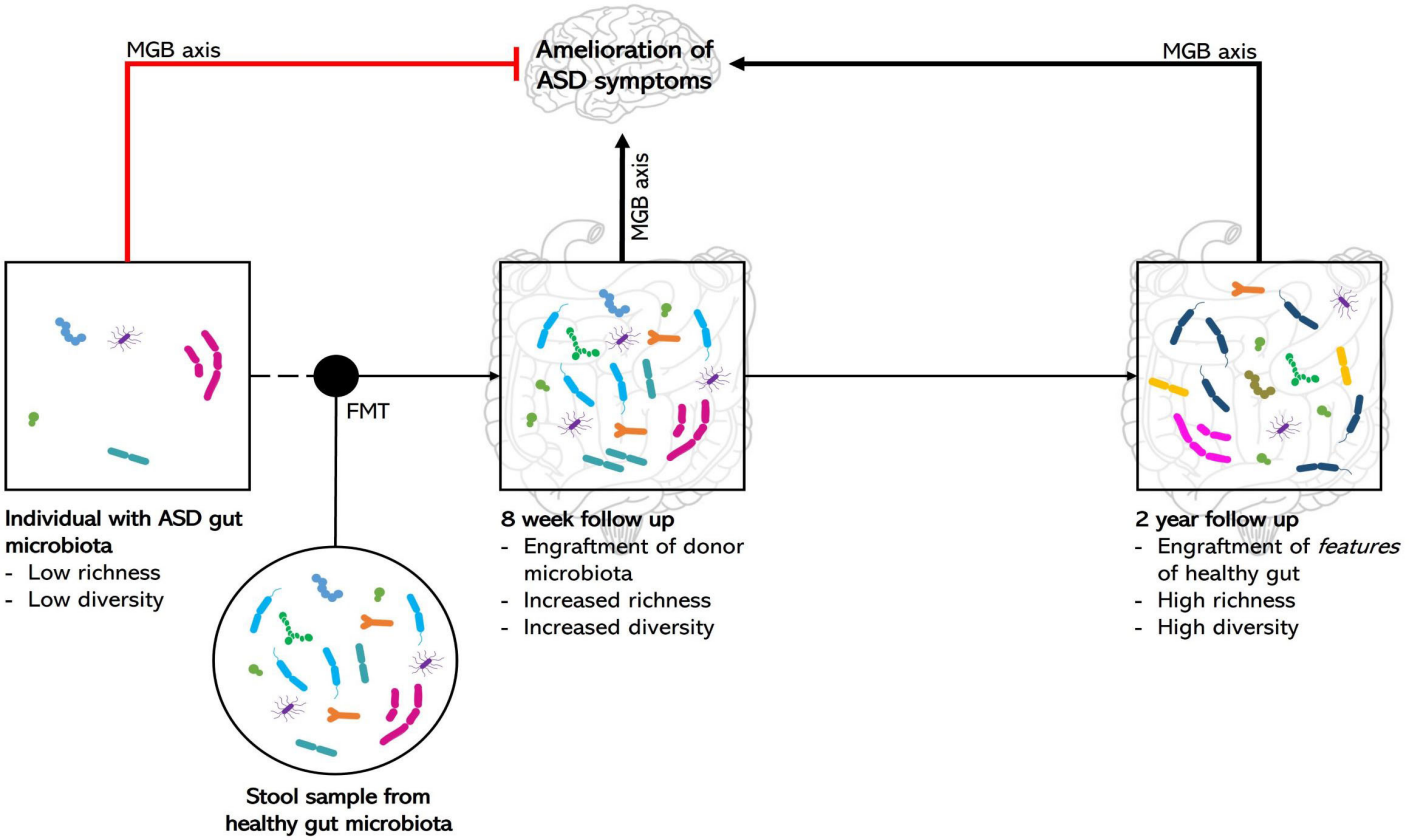
The proposed effects of FMT on gut microbiota composition from short-term and long-term follow-up studies. In the short-term follow-up study, FMT, using a TD donor sample, resulted in the engraftment of the donor microbiota so that it was indistinguishable from the donor. In the long-term follow-up study, there was only engraftment of the features of a healthy gut, such as high levels of richness and diversity and a high abundance of specific bacterial species, but the microbiota composition was no longer similar to that of the donor. Both the short-term and long-term restructuring of the gut microbiota as a result of FMT may cause amelioration of ASD symptoms due to the proposed link between the gut and neurocognitive health: the microbiota–gut–brain (MGB) axis. The information included in this figure has been obtained from (Chen et al., 2020; Kang et al., 2017; Kang et al., 2019; Li et al., 2021; Abujamel et al., 2022; Larsen and Claassen, 2018). ASD, autism spectrum disorder; MGB, microbiota-gut-brain; FMT, fecal microbial transplantation.

Both human and rodent studies show that treatment with FMT leads to an increase in microbial diversity and richness relative to the baseline in participants with ASD (Kang et al., 2017; Kang et al., 2019; Chen et al., 2020; Abujamel et al., 2022). This is of importance because research suggests that higher levels of gut microbiota diversity is considered healthier due to stronger microbiota integrity and functional redundancy, which helps to protect the gut from pathogenic bacteria (Larsen and Claassen, 2018). Furthermore, FMT was able to promote engraftment of donor microbiota so that participants’ microbiota composition was nearly indistinguishable from that of the TD donors 8 weeks post-intervention (Kang et al., 2017; Li et al., 2021). Interestingly, at the 2-year follow-up, the participants’ microbiota was again distinguishable from the donors, but the features of a healthy gut, such as a high level of bacterial density and a high abundance of *Prevotella*, were retained, as illustrated in **Figure 2** (Kang et al., 2019). This suggests that FMT leads to a long-term benefit in gut health by shifting the features of gut microbiota toward that of TD controls.

Specific differences in gut microbiota composition and abundance of species were unique to each study. For example, Kang et al. found that, following FMT, the relative abundances of *Bifidobacterium*, *Prevotella*, and *Desulfovibrio* were all significantly higher than baseline at both 8 weeks and 2 years post-intervention (Kang et al., 2017; Kang et al., 2019), whereas Chen et al. found that there was a “decrease in the relative abundance of *Prevotella_other* and a significant increase in the relative abundance of *Oscillospira*” (Chen et al., 2020). In rodent models, contrasting findings may be due to the different models used to induce ASD behaviors; using different chemical or immune-activated models will inherently change the structure of the gut microbiota, as shown by previous research (Coretti et al., 2017). In human studies, the variation in findings may be attributed to differences between the intestinal landscapes of both donors and FMT recipients. Li et al. found bacterial differences prior to treatment in participants who responded to FMT compared with those who saw no improvement post-intervention (Li et al., 2021). There is also evidence that indicates that the success of FMT depends on the microbial composition of donor samples, and there may be so-called “super-donors” who have the optimum microbial composition for a therapeutic intervention (Kump et al., 2018). This evidence combined suggests that there is a future where donors and recipients may be matched based on their gut microbiota to enable restoration of specific microbial deficits. Collection of useful clinical information, such as the initial microbiota composition, will help with the characterization of recipients and donors to reduce the variability of patient responses in the future and optimize targeting of donors.

### 3.4 Strengths and limitations of studies

To date, there has been no blinded randomized control trial (RCT) to investigate the effects of FMT on ASD symptoms. In order to reduce the possibility of the placebo effect and researcher bias, a placebo-controlled, double-blind RCT would be of value to the evidence base. The sample should be carefully considered as there is a well-documented sex bias in ASD diagnosis and research (Tillmann et al., 2018). All included studies report vastly higher proportions of male participants than female (see **Table 1**). Evidence shows that males and females present symptoms of ASD differently, so it is crucial that research into therapeutic options is carried out to reflect this (Harrop et al., 2018; Tillmann et al., 2018). Furthermore, the studies all used relatively small sample sizes of fewer than 60 participants: a larger sample size will increase the statistical power of any findings from a future RCT. Finally, the included studies only test up to the age of 18 years. As previously discussed, symptoms of ASD often persist into adulthood (Chamak and Bonniau, 2016). Therefore, it is important to consider whether the symptoms can be ameliorated in older individuals to reduce the significant negative outcomes they experience. There are several strengths of this review, including that it summarizes research studies that have shown FMT to be effective in alleviating symptoms of ASDs, while at the same time discussing limitations and future directions.

## 4 Conclusions and future work

Although the evidence presented here demonstrates the application of FMT in reducing the severity of ASD and GI symptoms in children and remodeling the gut microbiota, this review highlights the lack of robust RCTs for understanding the efficacy of this treatment. Some considerable gaps in the evidence base include the use of FMT as a therapeutic for children with ASD but no co-occurring GI symptoms, the effects of gender on the efficacy of FMT as a therapeutic for ASD, and whether FMT has the same ameliorative effects in adults and older populations with ASD.

FMT appears to be safe and well tolerated in a population with ASD, with few adverse events reported across all studies. Overall, the most effective treatment option appears to be a more intensive treatment regime of FMT, with no preference for administration routes. However, the optimum treatment regimen should be further investigated. In addition, there should be more investigation into the possibility of an optimum donor gut composition to improve the efficacy of FMT. There is mixed evidence regarding the use of preparation procedures prior to administration of FMT. Studies are limited to open-label designs and small samples and are heavily weighted toward male participants and children; hence, research is limited in generalizability. Future placebo-controlled RCTs using larger and more representative population samples are required to provide robust evidence for the optimum dose, treatment course, and use of preparation procedures. The impact of FMT on ASD sufferers needs to be further explored as it has been shown to alleviate ASD symptoms with potential benefits to patients and their families.

## Author contributions

RG conceptualized and wrote the manuscript. IN conceptualized and reviewed the manuscript, adding critical changes. All authors contributed to the article and approved the submitted version.
